# Low-grade intracranial meningioma with bilateral pulmonary metastases incidentally detected postpartum: a case report and review of the literature

**DOI:** 10.1186/s13256-021-03093-w

**Published:** 2021-10-14

**Authors:** Parviz Mardani, Arash Safarian, Anita Ashari, Sarina Pourjafar, Mohammad Hossein Anbardar, Negar Azarpira, Masoud Vafabin, Shahaboddin Yousefi

**Affiliations:** 1 Thoracic and Vascular Surgery Research Center, Shiraz University of Medical Sciences, Shiraz, Iran; 2 Department of Neurosurgery, Shiraz University of Medical Sciences, Shiraz, Iran; 3 Student Research Committee, Shiraz University of Medical Sciences, Shiraz, Iran; 4 Department of Pathology, Shiraz University of Medical Sciences, Shiraz, Iran; 5 Transplant Research Center, Shiraz University of Medical Sciences, Shiraz, Iran; 6 General Surgery Resident, Shiraz University of Medical Sciences, Shiraz, Iran; 7 Neurosurgery Resident, Shiraz University of Medical Sciences, Shiraz, Iran

**Keywords:** Meningioma, Extra-axial metastasis, Lung, Pregnancy, Case report

## Abstract

**Introduction:**

Meningiomas are the most commonly encountered intracranial tumors, usually showing indolent behavior. Extra-axial spreading and distant metastases are seldom detected in these tumors, and lung metastasis from a low-grade meningioma is a rare event.

**Case presentation:**

This case report aimed to present the clinical, imaging, and pathological features of a 37-year-old Caucasian pregnant woman with bilateral lung metastases incidentally detected during preoperative workup ahead of surgery for a primary intracranial meningioma. The possible metastatic routes and risk factors of dissemination to the pulmonary circulation were discussed as well.

**Conclusion:**

Metastasis must be considered in patients with intracranial meningiomas accompanied by venous sinus invasion and extension through the calvarium. Thorough paraclinical investigations are suggested in such cases.

## Background

Meningiomas are extra-axial tumors that are derived from the meningothelial cells of the arachnoid membrane. They are the most common types of primary brain tumor, and females are affected more than males, with a 2:1 ratio. Ionizing radiation and genetic syndromes such as neurofibromatosis type 2 (NF2) have been considered as the risk factors for meningiomas.

Meningiomas are mainly slow-growing. However, they can be aggressive or may even undergo malignant transformation in a small proportion of cases [1, 2]. According to the 2016 classification of central nervous system tumors by the World Health Organization (WHO), meningiomas are classified into three grades; that is, I, II (atypical), and III (malignant). It has been reported that, in the course of the disease, 0.1–1% of all primary meningiomas develop metastases, with the lung being the most frequent site of distant metastatic spread [1–3]. The present study aims to report the case of a pregnant patient with bilateral metastatic pulmonary lesions incidentally detected ahead of resection of the primary brain meningioma.

## Case presentation

**Fig. 1.**
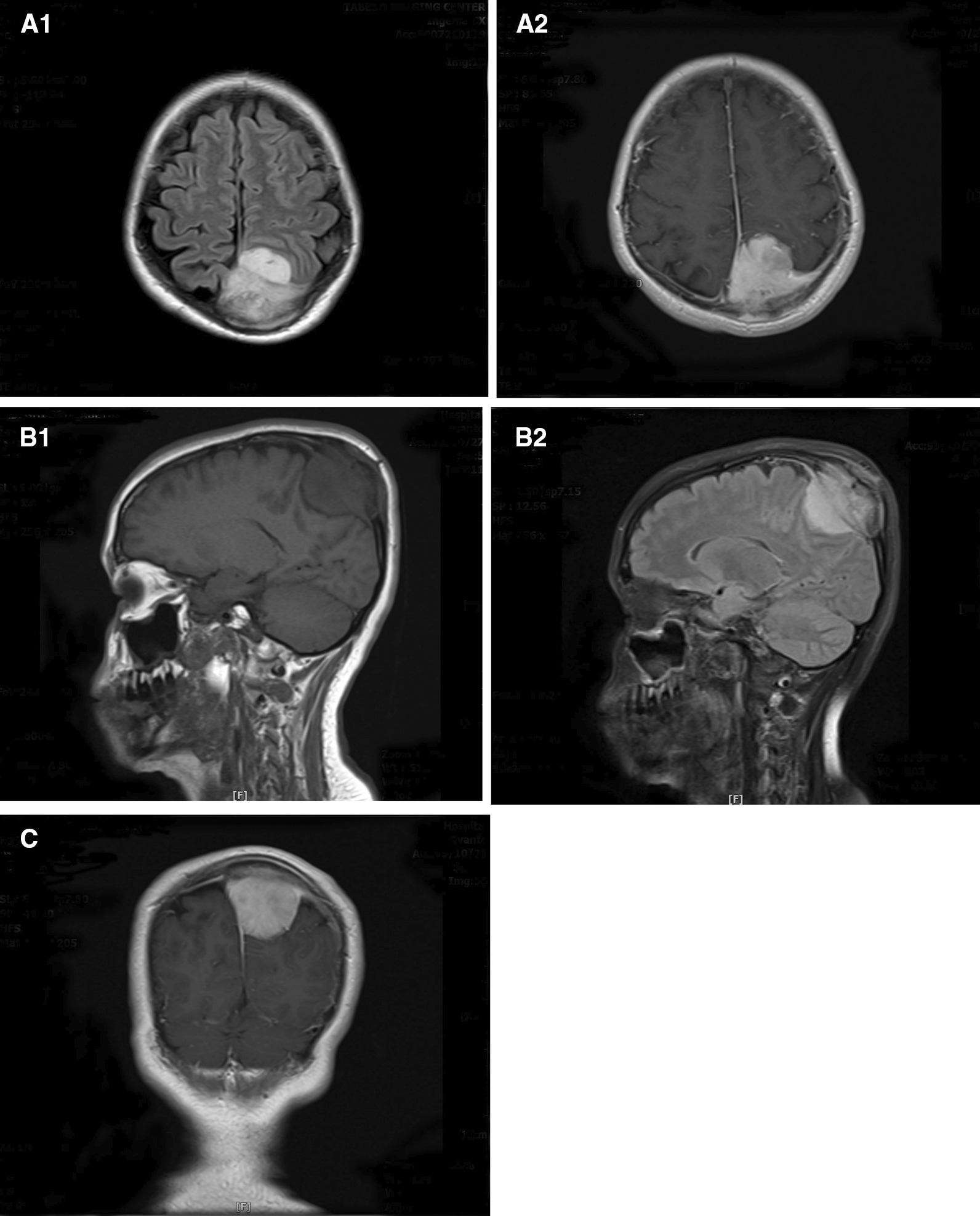
Preoperative magnetic resonance imaging of the patient with intracranial meningioma, showing a homogeneously enhancing tumor in the parasagittal region: (**A1**, **A2**) axial, (**B1**, **B2**) sagittal, and (**C**) coronal views

A 37-year-old Caucasian female presented at 18 weeks of gestation with the complaint of a slow-growing scalp mass. She had no history of headaches, blurred vision, nausea, or vomiting. In her past medical history, she had two pregnancies with no complications. On palpation, a well-defined round-to-oval mass was detected with hard consistency. However, the physical examination was unremarkable. Her full-term baby was delivered via cesarean section without any fetal or maternal complications. One month postpartum, the patient was visited by a general surgeon and a tissue biopsy was obtained from the scalp mass. The pathology result was in favor of a grade I meningioma with skull bone involvement. Hence, the patient was referred to the neurosurgery ward. Therein, a brain magnetic resonance imaging (MRI) was done, and an extra-axial 39 × 33 × 25 mm mass isointense in T1, hyperintense in T2, was noted high in the parafalcine region of the left parietal lobe. The mass had a dural tail appearance. Adjacent hyperostosis and enhancement of the adjacent involved calvarium in the high parietal scalp were mentioned as well. The findings were suggestive of an intraosseous meningioma with both intra- and extracranial components (Fig. 1).

The patient’s brain meningioma was operated in the neurosurgery department. Right frontoparietal craniotomy was performed, and the tumor was released from the brain tissue ahead of the gross total resection of the mass as well as the involved scalp. The postoperative pathology result of the brain mass was also in favor of grade I/III meningioma. The patient was discharged from the hospital with no complications and no adjuvant cranial radiotherapy.

**Fig. 2.**
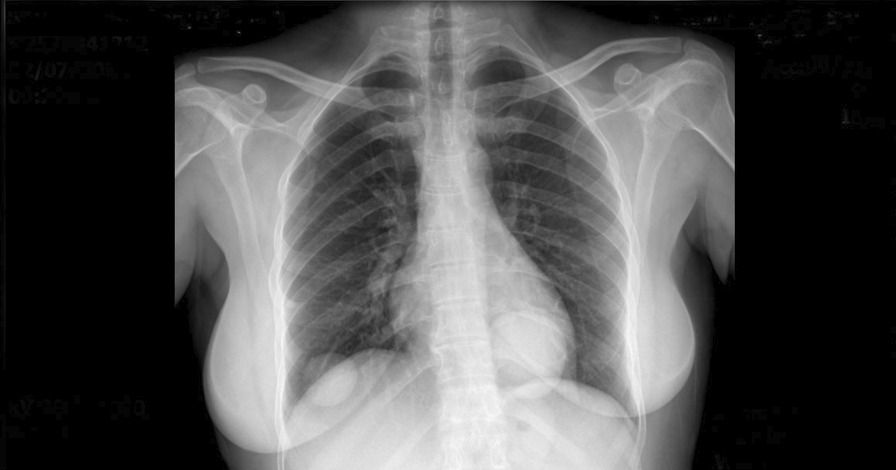
Chest X-ray showing bilateral intraparenchymal lung lesions

**Fig. 3.**
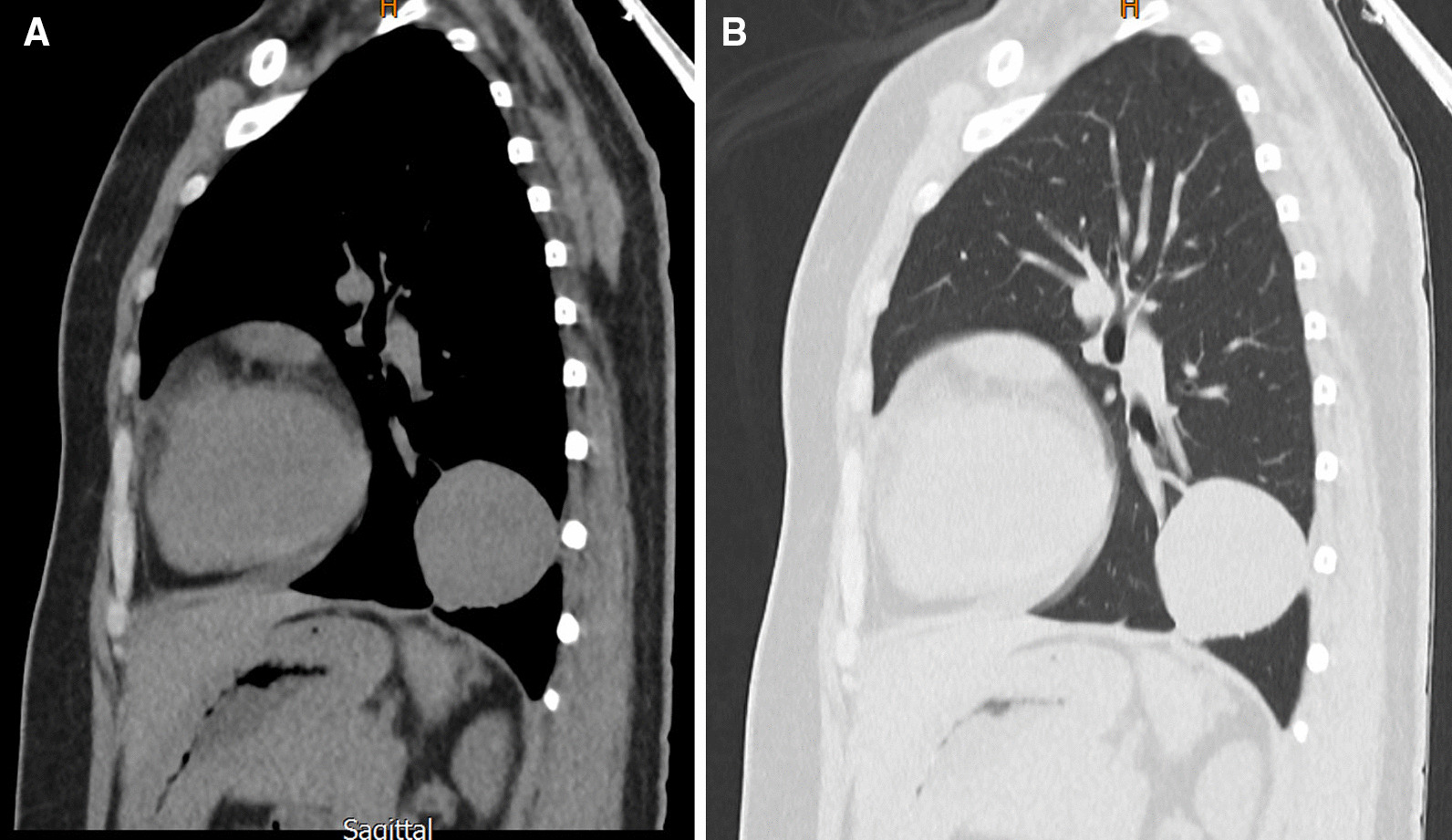
Chest CT scan of the lung mass (lateral view). Two well-defined lung masses are apparent

During the preoperative workup ahead of brain surgery, bilateral lung masses were detected as an incidental finding on chest X-ray and high-resolution computed tomography (HRCT) scan (Figs. 2, 3). There was no history of cough, chest pain, hemoptysis, or difficulty breathing. Heart and lung physical examinations were unremarkable. Preoperative laboratory evaluation including routine complete blood tests, electrocardiogram, and spirometry were unremarkable as well.

**Fig. 4.**
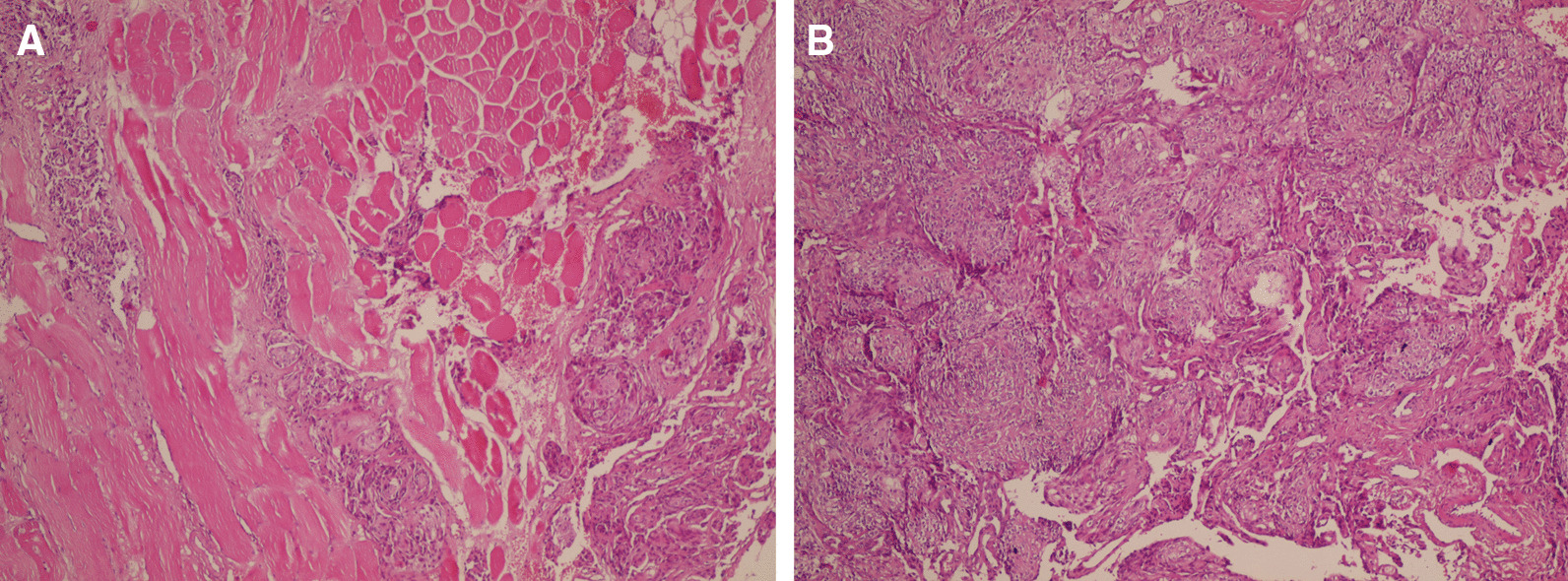
Histopathology of the resected brain mass and the pulmonary lesion showing tumoral cells with whorled appearance without atypia and mitotic figures compatible with meningioma grade I (**A**). The same pattern is obvious in the lung (**B**) (hematoxylin and eosin ×100)

**Fig. 5.**
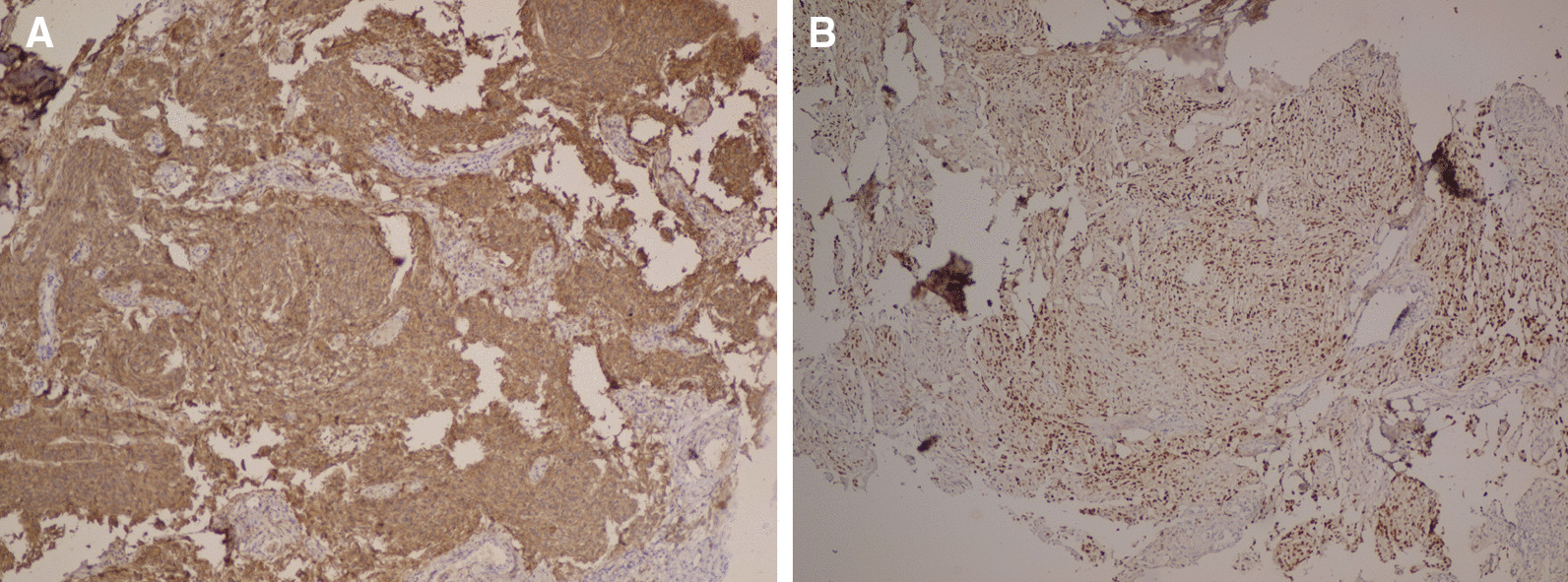
Immunohistochemical staining showing tumor cells positive for epithelial membrane antigen (**A**) and progesterone receptor (**B**) (×100)

A wedge resection of the left lobe lesion (4 × 5 cm) was performed along with a Tru-Cut biopsy of a pleural-based mass in the right lower pulmonary lobe. The histological feature of the lung tumor was the same as that of the brain mass, and the diagnosis of metastatic meningioma was confirmed by immunohistochemistry (IHC). Accordingly, the tumor cells were positive for EMA, PR, and Ki67 (low) and negative for P63, TTF1, and chromogranin (Figs. 4, 5). Bilateral lesions were resected through staged thoracotomy with no postoperative complications. The patient did not require radiotherapy and chemotherapy. She was followed up via brain and chest CT scans after 2 months, revealing satisfactory residual respiratory function and no evidence of pulmonary relapse.

## Discussion

Meningioma is a common primary slow-growing, intracranial, extra-axial neoplasm with attachment to the dura mater. It is composed of neoplastic meningothelial cells, and its usual growth rate is less than 1 cm/year, ranging from 0.03 to 2.62 cm/year [4]. Metastases may develop in less than 0.1% of patients, and lungs, liver, lymph nodes, and bones are the most common sites [5]. Pregnancy has been found to be associated with an increased incidence of symptomatic meningiomas in females, suggesting an increased tumor growth during this period [6]. On the other hand, attenuation of clinical symptoms and shrinkage of tumors have been reported during the postpartum period [2, 7].

Estrogen, progesterone, and prolactin levels are elevated during pregnancy, and the expression of the estrogen receptor (ER) and progesterone receptor (PR) has been found in a significant percentage of meningiomas [8]. These findings suggest that female sex hormone-induced cell proliferation is the simplest explanation for the growth of meningioma during pregnancy. On the other hand, exogenous estrogen therapy has not been associated with an increased risk of meningioma, and no change has been detected in the tumor size during the follicular phase of menstruation when estrogen levels are highest [8, 9].

Lusis *et al*. conducted a study on the pathology specimen of meningiomas during pregnancy and disclosed that the frequency of PR positivity was similar to that of the control group [10]. Although PR expression was found in 70–95% of meningiomas, the same level of expression was observed in tumoral tissues of children and males with minimal progesterone levels [10, 11]. Additionally, the grade of meningioma did not change during pregnancy [10].

Recently, Telugu *et al*. explored the expressions of ER and PR in meningioma specimens via IHC and assessed their correlations with gender, histological subtypes, and grade. The expression rates of ER and PR were 2% and 66%, respectively. No significant correlation was detected between the positivity of PR and the above-mentioned variables [12]. Therefore, the levels of sex hormones and expressions of receptors in tumoral tissues alone cannot justify the growth of meningiomas during pregnancy.

Another hypothesis that prevails to date is that meningioma growth is associated with vascular changes during pregnancy such as intratumoral hypervascularity and tissue edema [10]. The exact mechanism of edema is unclear, but an increase in the expression of aquaporin (a water channel protein) during pregnancy and a positive effect of progesterone on vascular dilatation have been suggested [10]. It seems that, during pregnancy, due to hemodynamic changes, a preexisting meningioma may present with the clinical symptoms of elevated intracranial pressure, including headache, nausea, and vomiting. After pregnancy, shrinkage of the tumoral tissue may occur and the mass size may decrease on its own.

Although meningiomas are more common among females, metastasis has not been reported to be more common in this population. For instance, multiple pulmonary metastases from preexisting intracranial meningiomas are rare in females. To the best of our knowledge, 33 such cases have been reported in the literature (Table 1) [1, 2, 7, 13–41]. The median age of the patients was 50 years (age range 26–91 years). In addition, the interval between the detection of the primary meningioma and the detection of lung metastases ranged from 9 to 19 years. In two cases, the lung mass was found concurrently with the primary brain tumor. Generally, lung metastases rarely cause symptoms such as cough and hemoptysis. In the present case, bilateral pulmonary nodules were found incidentally and the patient had no respiratory symptoms. The exact mechanism of multiple lung metastases from an intracranial tumor is not clear yet. Higher histological grades (II/III), venous sinus invasion, prior surgery for resection of the primary tumor, and tumor recurrence have been reported as the predictive factors for multiple lung metastases. Dissemination of tumor cells by hematogenous and lymphatic vessels or by cerebrospinal fluid (CSF) seeding have also been suggested as the underlying mechanisms of metastasis [42]. Tumor invasion to dural venous sinuses and cranial veins facilitate the hematogenous spread of tumoral cells to the pulmonary circulation [42]. The present case had a large mass with the invasion of the superior sagittal sinus and extension through the calvarium and scalp. Genetic abnormalities such as loss of heterozygosity at 9p, 1p, and 22q have also been considered the predictors of lung metastases [1].

**Table 1.** Demographic features, treatment and outcome of reported cases

Reference	Age, years	Interval, years	Location	Histology WHO grade	Treatment	Outcome
Aumann *et al*. [13]	45	5	Left frontal parasagittal	I	Total resection	NA
LeMay *et al*. [14]	56	10	NA	I	Partial resection	Died of disease 3 years after thoracotomy
Hishima *et al*. [15]	25	Prior to intracranial tumor	Right parietal region adjacent to the falx	I	Partial resection	NA
Murrah *et al*. [16]	53	10	Left frontal hemispheric convexity	NA	Partial resection	Alive with disease 2 years after thoracotomy
Adlakha *et al*. [17]	39	6	Left parietal parasagittal	II	Partial resection and gamma knife radiosurgery	Died of disease 10 years after initial presentation
Figueroa *et al*. [18]	50	5	Left cranial fossa	I	Total resection and radiotherapy for metastases	Alive with disease 32 years after radiotherapy
Travitzky *et al*. [19]	41	19	NA	III	Total resection and radiotherapy	No evidence of disease 6 months after doxil-induced regression of metastases
Erman *et al*. [7]	34	8	Left frontal-parasagittal	II	Partial resection and radiotherapy	Died of disease shortly after thoracotomy, radiotherapy, and chemotherapy of the metastasis
Psaras *et al*. [20]	65	15	Falx cerebri and superior sagittal sinus	I	Total resection and radiotherapy	No evidence of disease 12 months after thoracotomy
Alexandra *et al*. [21]	26	NA	Multiple supratentorial	III	Partial resection	NA
	84	NA	Right frontal	NA	Total resection	NA
	52	NA	Right frontal, left parietal, and right occipital	II	Partial resection	NA
Sabet *et al*. [22]	62	Concurrent	Left frontal	III	Partial resection and radiotherapy	NA
Nakayama *et al*. [23]	25	concurrent	Right parietal	I	Total resection	No evidence of disease 7 years after last surgery
Ocque *et al*. [24]	44	NA	NA	III	NA	NA
Frydrychowicz *et al*. [25]	45	5	Left frontal	II	Surgery and radiotherapy	NA
Dalle Ore *et al*. [26]	69	5.4	Cerebellum	III	No intervention	Alive after 5 months F/U
	75	9.2	Falx, parasagittal	II	No intervention	Alive after 1.5 months F/U
	64	10.8	Parasagittal	III	Hydroxyurea, EBRT	Alive after 13 months F/U
Vakil *et al*. [1]	91	1	Left frontoparietal	III	Radiotherapy with sunitinib	Alive after 13 months F/U
Sathirareuangchai *et al*. [27]	59	Concurrent	Left occipital convexity	I	Surgery	NA
Wang *et al*. [2]	54		Left middle cranial	I	Surgery	NA
Som *et al*. [38]	53	5	Right sphenoid wing	I	Surgery	NA
Kodama *et al*. [33]	61	19	Cerebellar	I	Surgery	NA
Tao *et al*. [40]	32	18	Right frontal	I	Surgery	NA
Shin *et al*. [37]	53	9	Left frontal	I	Surgery	NA
Tworek *et al*. [41]	50	6	Left frontal	I	Surgery	NA
Baisden *et al*. [29]	71	13	Right middle cranial fossa	II	Surgery	NA
Kovoor *et al*. [34]	40	2	Left parietal	I	Surgery	NA
Pramesh *et al*. [36]	29	9	Right occipital	I	Surgery	NA
Fabi *et al*. [31]	57	1	Right frontal	III	Surgery	NA
Asioli *et al*. [28]	58	12	NA	I	Surgery	NA
Brennan *et al*. [30]	74	22	Parasagittal	II	Surgery	NA
Kanzaki *et al*. [32]	67	15	NA	II	Surgery	NA
Lambertz *et al*. [35]	65	12	Right frontal	II		
Tao *et al*. [39]	51	1	Right lateral ventricle	III	Surgery	NA
Kansaki [32]	67	15	NA	II	Surgery	Alive
Current case	34	Concurrent	Left parafalcine	I	Surgery	Alive

*NA* not available

Surgical resection is the current standard of care for primary low-grade meningiomas. For high-grade lesions, surgical resection is combined with adjuvant radiotherapy to prevent local recurrence. Thoracotomy to surgically excise the lung mass is the treatment of choice for metastatic lung masses. Due to the rarity of metastatic meningiomas, few clinical trials have been performed and no standard treatment is available. Previous studies assessed the efficacy of hydroxyurea, external beam radiation, and sunitinib in treatment of high-grade metastatic cases [43, 44].

## Conclusion

In patients with intracranial meningiomas accompanied by venous sinus invasion and extension through the calvarium, metastasis must be considered and thorough paraclinical investigations are suggested. From a practical point of view, these seemingly benign lesions may not be as they appear, which emphasizes the importance of follow-up for such lesions beyond the scope of neurosurgery.

## Data Availability

No additional datasets were used for the creation of this manuscript. All information is available from the standard documentation in the patient’s electronic medical record.
